# A Systematic Review and Meta-Analysis on the Association between Hypertension and Tinnitus

**DOI:** 10.1155/2015/583493

**Published:** 2015-12-31

**Authors:** Pan Yang, Wenjun Ma, Yiqing Zheng, Haidi Yang, Hualiang Lin

**Affiliations:** ^1^School of Public Health, Sun Yat-sen University, Guangzhou, China; ^2^Guangdong Provincial Institute of Public Health, Guangdong Provincial Center for Disease Control and Prevention, Guangzhou, China; ^3^Department of Otolaryngology, Sun Yat-sen Memorial Hospital, Sun Yat-sen University, Guangzhou, China

## Abstract

Hypertension has been suggested to be one possible risk factor of tinnitus, but the association between hypertension and tinnitus remains uncertain. The authors performed a meta-analysis of the existing studies on the association between hypertension and tinnitus. We performed literature search of studies using SinoMed, CNKI, WanFang, PubMed, Scopus, Web of Science, and Google Scholar. Studies reported the odds ratio and 95% confidence interval (CI) (or provided sufficient information for calculation) of the association between hypertension and tinnitus were included. A total of 19 eligible studies with 20 effect estimates were used in this study. They included 63,154 participants with age ranging from 14 to 92. The pooled OR, which was pooled using a random effects model, was 1.37 (95% CI: 1.16 to 1.62). There was no evidence of publication bias (*p* = 0.11 for Begg's test, *p* = 0.96 for Egger's test). By meta-regression, we found that study design may be one possible factor of heterogeneity. Sensitivity analysis found that the result was stable. This study suggests that hypertension might be one risk factor of tinnitus, and hypertension prevention and control might be helpful in preventing tinnitus.

## 1. Introduction

Tinnitus is the perceived sensation of sound in the absence of a corresponding external acoustic stimulus [1]. It is a very bothersome symptom for many patients as it can affect the physical and mental health in different degree. The patients suffering from serious tinnitus may even commit suicide [2].

Hypertension has been suggested as one potential risk factor of tinnitus in some studies, but some other studies showed different results. Thirunavukkarasu and Geetha's retrospective study [3] showed that hypertension and giddiness were high risk factors for the occurrence of tinnitus. Nondahl et al.'s study [4] of ten-year incidence of tinnitus showed that hypertension was not associated with the incidence of tinnitus. Negrila-Mezei et al.'s case-control study [5] based on 471 ear-nose-throat department patients showed that hypertension was significantly associated with tinnitus, but de Moraes Marchiori's case-control study [6] did not find any significant association between hypertension and tinnitus. Some cross-sectional surveys [7, 8] showed that hypertension was a risk of tinnitus, but some other cross-sectional surveys [9, 10] did not find any significant association. It is important to elucidate and quantify the association between hypertension and tinnitus. If the association holds, prevention and control of hypertension should then be included in the prevention measures of tinnitus.

In this study, we provided a meta-analysis of the tinnitus risk associated with hypertension. We also performed a meta-regression to examine possible sources of heterogeneity between the studies and examined the influence of single study on the overall meta-estimate.

## 2. Methods

### 2.1. Literature Search

Eligible studies were searched via databases. The databases included SinoMed, CNKI, WanFang, PubMed, Scopus, Web of Science, and Google Scholar. Search strategies used subject headings and key words and did not have language and time restrictions. The presence of tinnitus was defined as answering “yes” to the question “In the past 12 months, have you ever heard a sound (buzzing, hissing, ringing, humming, roaring, machinery noise) originating in your ears?” or similar question in different phrase. Hypertension (high blood pressure) was defined as a systolic blood pressure (SBP) ≥ 140 mmHg or diastolic blood pressure (DBP) ≥ 90 mmHg or reported use of antihypertensive medication [11].

The studies were identified by combining the term “tinnitus” with several terms, such as hypertension, blood pressure, prevalence, risk factors, epidemiology, and characterization, which indicated that the study might provide the relevant information on the association between hypertension and tinnitus, such as OR (odds ratio) and 95% confidence intervals (95% CIs) or relevant information to calculate OR and 95% CIs. We also examined reference lists of the all identified studies and reviewed the cited literatures to identify any other relevant studies.

### 2.2. Inclusion and Exclusion Criteria

Studies were included in the current meta-analysis if they provided the information to examine the association between hypertension and tinnitus (OR and 95% CIs or relevant data to calculate OR and 95% CIs). Studies which reported one specific type of tinnitus (such as left ear tinnitus and pulsatile tinnitus) were excluded.

### 2.3. Meta-Analysis

To estimate the quantitative relationship between hypertension and tinnitus, we obtained estimates of the OR and 95% CIs from relevant studies. We used a random-effects model to combine estimates from the identified studies, which allowed between-study heterogeneity to contribute to the variance [12]. We assessed homogeneity of ORs with *I* ^2^ value which represented the estimated percent of total variance that could be explained by between-study heterogeneity [13]. Publication bias was assessed using Begg's test [14] and Egger's test [15]. Begg's funnel plot and Egger's publication bias plot were created to provide a visual investigation of possible publication bias. Meta-regression was used to explore whether the inconsistency in results across individual studies could be explained by variations in publication year, region (American and others), sample size, study design (case-control study and cross-sectional study), and confounder adjustment (the sets of potential confounders for which adjustment was made varied by study; we just assessed the difference between studies with adjusted ORs and studies with crude ORs) [16]. We performed both univariate and multivariate meta-regression analysis. Finally, we performed sensitivity analysis to examine the influence of individual studies, in which the meta-analysis estimates were derived by omitting one study at a time. The whole analyses were conducted using STATA software (v12.1).

## 3. Results

**Figure 1:**
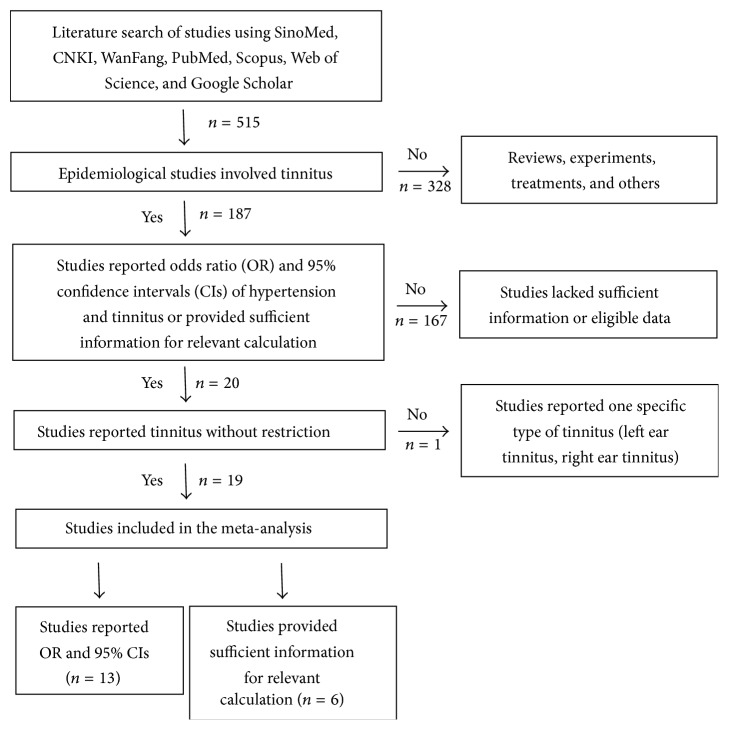
Flow diagram on the search process.

Of a total of 515 studies identified from the search strategy, 187 were epidemiological studies and 328 studies were literature reviews, experimental studies, clinical treatments, or others and were excluded from the analysis. We chose 20 studies reporting OR and 95% CIs of the association between hypertension and tinnitus or providing sufficient data for relevant calculation. Of the 20 studies, one study reported one specific type of tinnitus (left ear tinnitus, right ear tinnitus) and was excluded. Finally, we included a total of 19 studies which provided suitable information for the subsequent analysis (Figure 1).

We identified 3 case-control studies and 16 cross-sectional studies. Twenty population samples from 19 studies (Fujii et al.'s study [8] provided both men and women information) provided sufficient data for a meta-analysis. Details of the included studies were summarized in Table 1. Overall, this study included 63,154 participants from Italy, South Korea, China, Nigeria, USA, Brazil, Japan, Romania, Turkey, Australian, and Chile, the age of the subjects ranged from 14 to 92 years, sample size of the studies ranged between 120 and 14,178, and OR ranged between 0.73 (95% CI: 0.35 to 1.53) and 12.14 (95% CI: 5.04 to 29.23).

**Figure 2:**
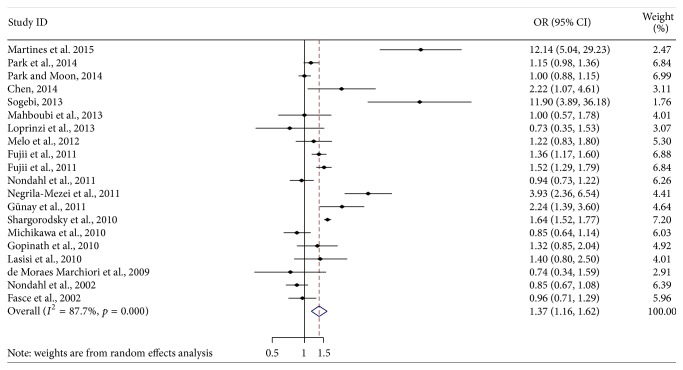
Forest plot of the associations between hypertension and tinnitus (OR and 95% CI indicate odds ratio and 95% confidence interval).

Figure 2 showed the forest plot of 20 effect estimates from 19 studies. Of them, eight studies showed a significant positive association between hypertension and tinnitus. The overall pooled OR was 1.37 (95% CI: 1.16 to 1.62).

**Figure 3:**
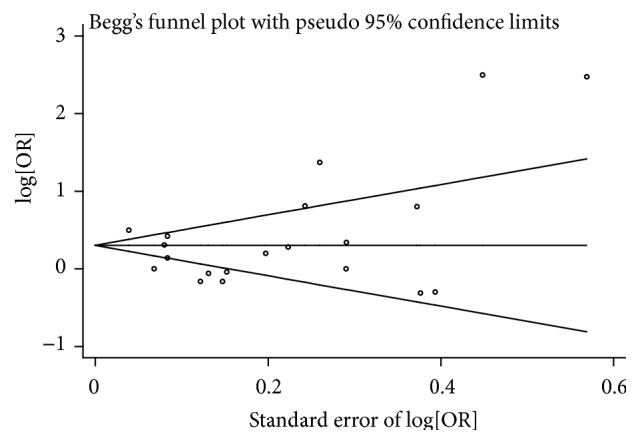
Begg's funnel plot for meta-analysis.

**Figure 4:**
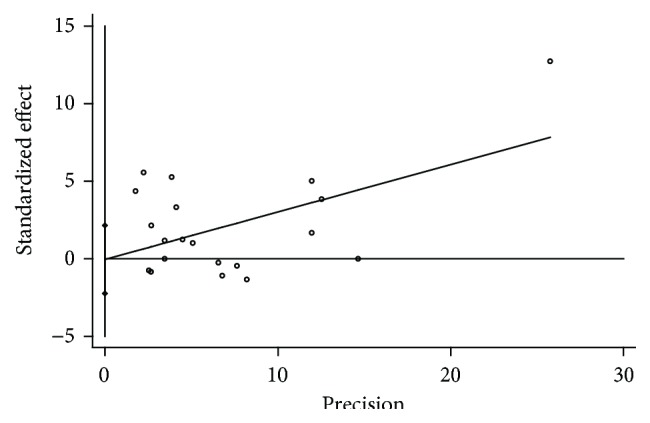
Egger's publication bias plot for meta-analysis.

Publication bias was not detected by Begg's test (*p* = 0.11) (Figure 3) and Egger's test (*p* = 0.96) (Figure 4). The heterogeneity test was significant (*Q* = 155.06, *p* < 0.001, *I* ^2^ = 87.7%, and Tau-squared = 0.103). Meta-regression (*I* ^2^ = 85.1%, Tau-squared = 0.090, and adjusted *R*-square = 73.6%) showed that, among various variables, study design was one significant contributor (*p* = 0.002) (Table 2).

**Figure 5:**
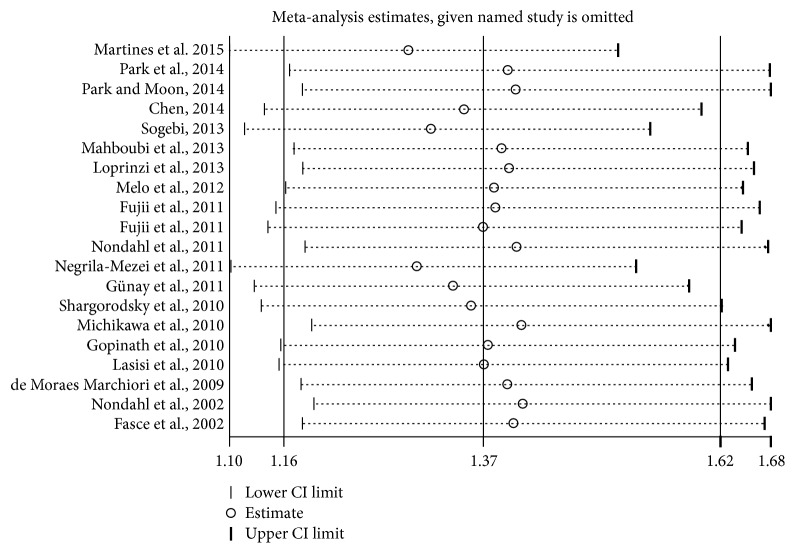
Sensitivity analysis.

The sensitivity analysis indicated that the omission of any of the studies led to changes in estimates between 1.29 (95% CI: 1.10 to 1.51) and 1.41 (95% CI: 1.19 to 1.68) (Figure 5).

## 4. Discussion

Tinnitus involves a large proportion of the general population and affects the quality of life and work efficiency. Most of tinnitus prevalence studies in Western Europe and USA have reported prevalence rates between 10% and 15% in the adult population [30]. For example, the largest study (*n* = 48313), which was undertaken as part of the National Study of Hearing in England, showed a prevalence of 10.1% among adults, with 2.8% of respondents describing it as moderately annoying, 1.6% as severely annoying, and 0.5% at a level severely affecting their normal life [31]. The mechanism of occurrence of tinnitus remains largely unknown; although several treatment strategies for tinnitus patients have been proposed, such as the tinnitus masking technique, pharmacological therapy, and surgery, no single effective cure exists for tinnitus [32]. It is necessary and important to study the risk factors of tinnitus, which will be helpful for formulating specific prevention measures for its prevention. This study provided a quantitative meta-analysis of the association between hypertension and tinnitus. The pooled OR was 1.37 (95% CI: 1.16 to 1.62), which supports that hypertension is significantly associated with tinnitus.

Hypertension has been suggested as one potential risk factor of tinnitus in some studies. There are some studies about hypertension in patients with tinnitus; for example, Nowak et al.'s study [33] included 1200 patients getting treated in the Laryngological Rehabilitation Centre in Poznań due to tinnitus, in the examined group 34% suffered from systemic diseases, and among them the highest percentage (47%) suffered from hypertension; Sogebi's study [20] showed that, among 79 patients having complaints of tinnitus, 15.2% were suffering from hypertension. There are also some studies about tinnitus in patients with hypertension; for instance, Borghi et al.'s study [34] showed that 17.6% patients (aged 18 to 75 years, with uncontrolled hypertension) reported occasional or prolonged spontaneous tinnitus and suggested that systemic blood pressure might have played a prominent role in some tinnitus patients; Chávez-Delgado et al. conducted a cross-sectional study of 385 patients with hypertension, type 2 diabetes mellitus, and dyslipidemia with symptoms of hearing loss, vertigo, and tinnitus; the prevalence of tinnitus was 32% in patients with hypertension [35].

The pathophysiological mechanisms of tinnitus are still not clear. There are several possible mechanisms including increased spontaneous firing rate of neurons in the central auditory system, increased neural synchrony in the firing pattern across neurons in primary auditory cortex, and map reorganisation in the auditory modality [30]. The mechanisms underlying the relationship between hypertension and tinnitus are poorly understood. There are animal experiments [36, 37] indicating that hypertension may induce the occurrence of tinnitus or aggravate preexisting tinnitus through two principle mechanisms, high blood pressure might cause damage to the cochlear microcirculation, and diverse antihypertensive drugs might lead to ototoxicity. Tachibana et al. [37] suggested that the primary target of hypertensive damage in the rat cochlea is the stria vascularis, which feeds the organ of Corti. Przewoźny et al. [38] suggested that the subclinical damage to the stria vascularis includes the decrease in the cochlear oxygen partial pressure and disturbance of the ionic K^+^ recycling. Borghi et al. [34] suggested that the presence of tinnitus is the consequence of toxic damage of the labyrinth in most patients, which means that the onset of tinnitus might be an adverse event in patients treated with antihypertensive drugs. Another possibility pointed to the fact that when antihypertensive action exceeded the desired effects, subsequent abrupt hypotension would lead to cochlear hypoperfusion and circulatory impairment, which could disturb the sensorineural response, leading to the onset of tinnitus [34]. Clinical evidence also indicated an association between hypertension and tinnitus, which reinforces the hypothesis that alterations in the cochlear microcirculation, as causal or adjuvant factors in tinnitus pathophysiology, occur [39].

There is widespread recognition that consistency between research centers in the ways that patients with tinnitus are assessed and outcomes following interventions are measured would facilitate more effective cooperation and more meaningful evaluations and comparisons of outcomes [40]. But many studies still have methodological drawbacks, especially with production of an unambiguous definition of tinnitus and phrasing of appropriate epidemiological questions, which may be one of the main causes for the heterogeneity in the present study [30]. The other cause for heterogeneity of this research may include socioeconomic condition, ethnic differences, and public health service. Although the meta-analysis detected significant heterogeneity between studies, further sensitivity analyses and the exclusion of publication bias are in favor of a similar effect across the populations.

Hypertension is an important public health concern worldwide. Globally, the overall prevalence of hypertension in adults aged 18 years and over was around 22% in 2014 [41]. Kearney et al.'s study [42] estimated that 29% (about 1.56 billion) of the world's adult population would have hypertension by 2025. Since hypertension is one of the high incidence chronic diseases and has large numbers of patients, we must pay attention to the fact that tinnitus may be caused by hypertension and consider the ototoxicity of drugs for hypertension patients, which means that we may make a choice for hypertension medication to prevent tinnitus.

A few limitations should be noted. First, the included studies for this meta-analysis varied in the degree of controlling for potential confounders, such as age, gender, ethnicity, income, smoking, ear infecting, noise exposure, BMI, anemia, and hearing loss, only a few of the ORs were adjusted, and the adjusted ORs did not adjust for all the same factors. Second, the study design was either cross-sectional or case-control study; therefore it cannot determine temporal sequence and causality [43]. Third, our study did not investigate any causal mechanism of tinnitus and simply did quantitative analysis of the association between hypertension and tinnitus, this association may not be the direct causal role, and further studies are needed.

In conclusion, this meta-analysis suggests that hypertension might be a risk factor of tinnitus and should be considered in its prevention strategy.

## Figures and Tables

**Table 1:** Description of the study populations included in the meta-analysis (*n* = 63154).

First author	Year	Country	Study design	Sample size (*n*)	Age (years)
Martines [17]	2015	Italy	Case-control	120	14–85
Park [18]	2014	South Korea	Cross-sectional	5140	≥40
^*∗*^Park [9]	2014	South Korea	Cross-sectional	10061	20–97
Chen [19]	2014	China	Case-control	204	NA
Sogebi [20]	2013	Nigeria	Cross-sectional	127	≥41
^*∗*^Mahboubi [21]	2013	American	Cross-sectional	3520	12–19
^*∗*^Loprinzi [22]	2013	American	Cross-sectional	473	70–85
^*∗*^Melo [23]	2012	Brazil	Cross-sectional	498	≥60
^*∗*^Fujii [8]	2011	Japan	Cross-sectional	6450 (men)	45–79
^*∗*^Fujii [8]	2011	Japan	Cross-sectional	7973 (women)	45–79
^*∗*^Nondahl [24]	2011	American	Cross-sectional	3267	21–84
Negrila-Mezei [5]	2011	Romania	Case-control	471	≥60
^*∗*^Günay [7]	2011	Turkey	Cross-sectional	879	18–64
Shargorodsky [25]	2010	American	Cross-sectional	14178	NA
Michikawa [26]	2010	Japan	Cross-sectional	1286	≥65
Gopinath [27]	2010	Australian	Cross-sectional	1214	NA
^*∗*^Lasisi [10]	2010	Nigeria	Cross-sectional	1302	≥65
de Moraes Marchiori [6]	2009	Brazil	Cross-sectional	154	45–64
Nondahl [28]	2002	American	Cross-sectional	3737	48–92
Fasce [29]	2002	Chile	Cross-sectional	2100	NA

^*∗*^OR was adjusted; NA: not available.

**Table 2:** Parameter estimation of meta-regression.

Variable	Univariate		Multivariate	
	Coefficient	95% CI	Coefficient	95% CI
Publication year	0.07	(−0.02, 0.16)	0.04	(−0.06, 0.13)
Region	−0.49	(−1.23, 0.25)	−0.21	(−0.86, 0.43)
Sample size	−3.46*e* − 5	(−12.14*e* − 5, 5.23*e* − 5)	7.06*e* − 6	(−6.71*e* − 5, 8.12*e* − 5)
Study design	1.28	(0.54, 2.02)	1.10	(0.07, 2.14)
Confounder adjustment	0.34	(−0.32, 1.01)	0.10	(−0.53, 0.73)

95% CI indicates 95% confidence interval.
